# Supplementary material for: Parent-progeny imputation from pooled samples for cost-efficient genotyping in plant breeding
Source: PLoS One. 2017 Dec 22;12(12):e0190271. doi: 10.1371/journal.pone.0190271 (PMC5741258; doi:10.1371/journal.pone.0190271)
Supplement: S1 File — The recurrent and donor parents of the first DH are R1 and D1, respectively. Those of the second DH are R2 and D2. The recombination frequency between locus k and k − 1 is rk. (PDF) [file pone.0190271.s001.pdf]

$$
\begin{array}{c|cccc}
 & R_1 - R_2 & R_1 - D_2 & D_1 - R_2 & D_1 - D_2 \\
\hline
R_1 - R_2 & \left(\frac{2}{3}\left[1 + \frac{1}{2}(1 - r_k)^2\right]\right)^2 & \frac{2}{3}(1 + \frac{1}{2}(1 - r_k)^2) * \frac{1}{3}(2r_k - r_k^2) & \frac{2}{3}(1 + \frac{1}{2}(1 - r_k)^2) * \frac{1}{3}(2r_k - r_k^2) & \left(\frac{1}{3}(2r_k - r_k^2)\right)^2 \\
R_1 - D_2 & \frac{2}{3}(1 + \frac{1}{2}(1 - r_k)^2) * (2r_k - r_k^2) & \frac{2}{3}(1 + \frac{1}{2}(1 - r_k)^2) * (1 - r_k)^2 & \frac{1}{3}(2r_k - r_k^2)^2 & \frac{1}{3}(2r_k - r_k^2) * (1 - r_k)^2 \\
D_1 - R_2 & (2r_k - r_k^2) * \frac{2}{3}(1 + \frac{1}{2}(1 - r_k)^2) & \frac{1}{3}(2r_k - r_k^2)^2 & (1 - r_k)^2 * \frac{2}{3}(1 + \frac{1}{2}(1 - r_k)^2) & (1 - r_k)^2 * \frac{1}{3}(2r_k - r_k^2) \\
D_1 - D_2 & (2r_k - r_k^2)^2 & (2r_k - r_k^2) * (1 - r_k)^2 & (1 - r_k)^2 * (2r_k - r_k^2) & (1 - r_k)^4
\end{array}
$$

Transition matrix for a pool of two DH derived from a BC_1 generation The recurrent and donor parents of the first DH are R_1 and D_1, respectively. Those of the second DH are R_2 and D_2. The recombination frequency between locus k and $k-1$ is r_k.
