# Supplementary material for: Parent-progeny imputation from pooled samples for cost-efficient genotyping in plant breeding
Source: PLoS One. 2017 Dec 22;12(12):e0190271. doi: 10.1371/journal.pone.0190271 (PMC5741258; doi:10.1371/journal.pone.0190271)
Supplement: S2 File — Parent-progeny imputation is carried out for four genetically linked loci L1, L2, L3 and L4 for a DNA pool of three DH individuals (P1, P2, P3) from three biparental populations (I1 × I2, I3 × I4, I5 × I6). (PDF) [file pone.0190271.s002.pdf]

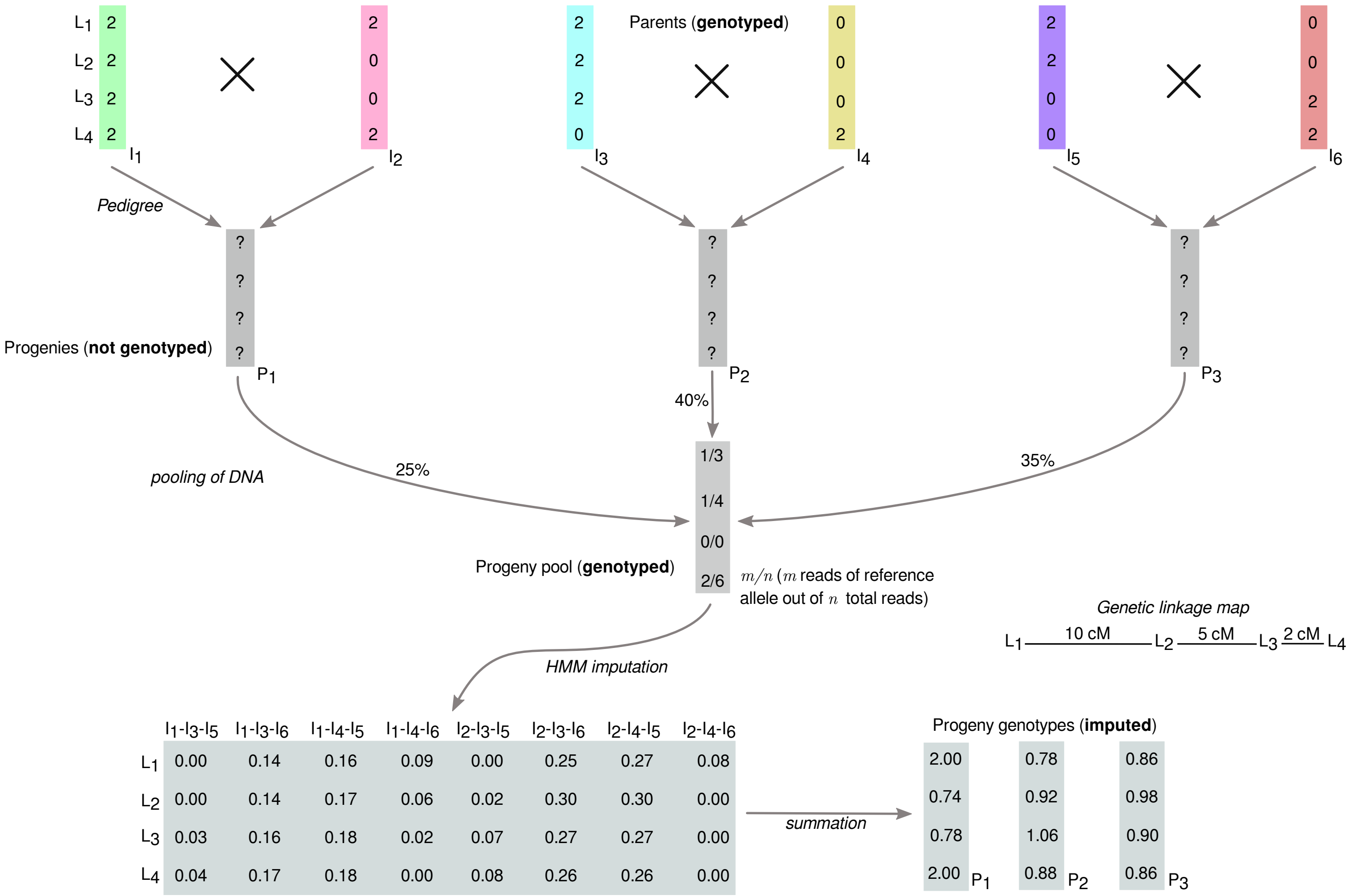

Figure 1. Example of parent-progeny imputation from a pool of three F_1 derived DH Parent-progeny imputation is carried out for four genetically linked loci L_1, L_2, L_3 and L_4 for a DNA pool of three DH individuals (P_1, P_2, P_3) from three biparental populations ($I_1 \times I_2$, $I_3 \times I_4$, $I_5 \times I_6$).

	$I_1-I_3-I_5$	$I_1-I_3-I_6$	$I_1-I_4-I_5$	$I_1-I_4-I_6$	$I_2-I_3-I_5$	$I_2-I_3-I_6$	$I_2-I_4-I_5$	$I_2-I_4-I_6$
$I_1-I_3-I_5$	$(1-r_k)^3$	$r_k(1-r_k)^2$	$r_k(1-r_k)^2$	$r_k^2(1-r_k)$	$r_k(1-r_k)^2$	$r_k^2(1-r_k)$	$r_k^2(1-r_k)$	r_k^3
$I_1-I_3-I_6$	$r_k(1-r_k)^2$	$(1-r_k)^3$	$r_k^2(1-r_k)$	$r_k(1-r_k)^2$	$r_k^2(1-r_k)$	$r_k(1-r_k)^2$	r_k^3	$r_k^2(1-r_k)$
$I_1-I_4-I_5$	$r_k(1-r_k)^2$	$r_k^2(1-r_k)$	$(1-r_k)^3$	$r_k(1-r_k)^2$	$r_k^2(1-r_k)$	r_k^3	$r_k(1-r_k)^2$	$r_k^2(1-r_k)$
$I_1-I_4-I_6$	$r_k^2(1-r_k)$	$r_k(1-r_k)^2$	$r_k(1-r_k)^2$	$(1-r_k)^3$	r_k^3	$r_k^2(1-r_k)$	$r_k^2(1-r_k)$	$r_k(1-r_k)^2$
$I_2-I_3-I_5$	$r_k(1-r_k)^2$	$r_k^2(1-r_k)$	$r_k^2(1-r_k)$	r_k^3	$(1-r_k)^3$	$r_k(1-r_k)^2$	$r_k(1-r_k)^2$	$r_k^2(1-r_k)$
$I_2-I_3-I_6$	$r_k^2(1-r_k)$	$r_k(1-r_k)^2$	r_k^3	$r_k^2(1-r_k)$	$r_k(1-r_k)^2$	$(1-r_k)^3$	$r_k^2(1-r_k)$	$r_k(1-r_k)^2$
$I_2-I_4-I_5$	$r_k^2(1-r_k)$	r_k^3	$r_k(1-r_k)^2$	$r_k^2(1-r_k)$	$r_k(1-r_k)^2$	$r_k^2(1-r_k)$	$(1-r_k)^3$	$r_k(1-r_k)^2$
$I_2-I_4-I_6$	r_k^3	$r_k^2(1-r_k)$	$r_k^2(1-r_k)$	$r_k(1-r_k)^2$	$r_k^2(1-r_k)$	$r_k(1-r_k)^2$	$r_k(1-r_k)^2$	$(1-r_k)^3$

Transition matrix for a pool of three F_1 derived DH The recombination frequency between locus k and $k-1$ is r_k.

	$I_1-I_3-I_5$	$I_1-I_3-I_6$	$I_1-I_4-I_5$	$I_1-I_4-I_6$	$I_2-I_3-I_5$	$I_2-I_3-I_6$	$I_2-I_4-I_5$	$I_2-I_4-I_6$
$m=0$	0.00	0.05	0.07	0.38	0.02	0.21	0.26	1.00
$m=1$	0.00	0.14	0.17	0.34	0.07	0.29	0.31	0.00
$m=2$	0.00	0.24	0.26	0.19	0.19	0.26	0.24	0.00
$m=3$	0.00	0.31	0.29	0.07	0.34	0.17	0.14	0.00
$m=4$	1.00	0.26	0.21	0.02	0.38	0.07	0.05	0.00

Emission matrix for a pool of three F_1 derived DH
