# Supplementary material for: Parent-progeny imputation from pooled samples for cost-efficient genotyping in plant breeding
Source: PLoS One. 2017 Dec 22;12(12):e0190271. doi: 10.1371/journal.pone.0190271 (PMC5741258; doi:10.1371/journal.pone.0190271)
Supplement: S1 Fig — (PDF) [file pone.0190271.s004.pdf]

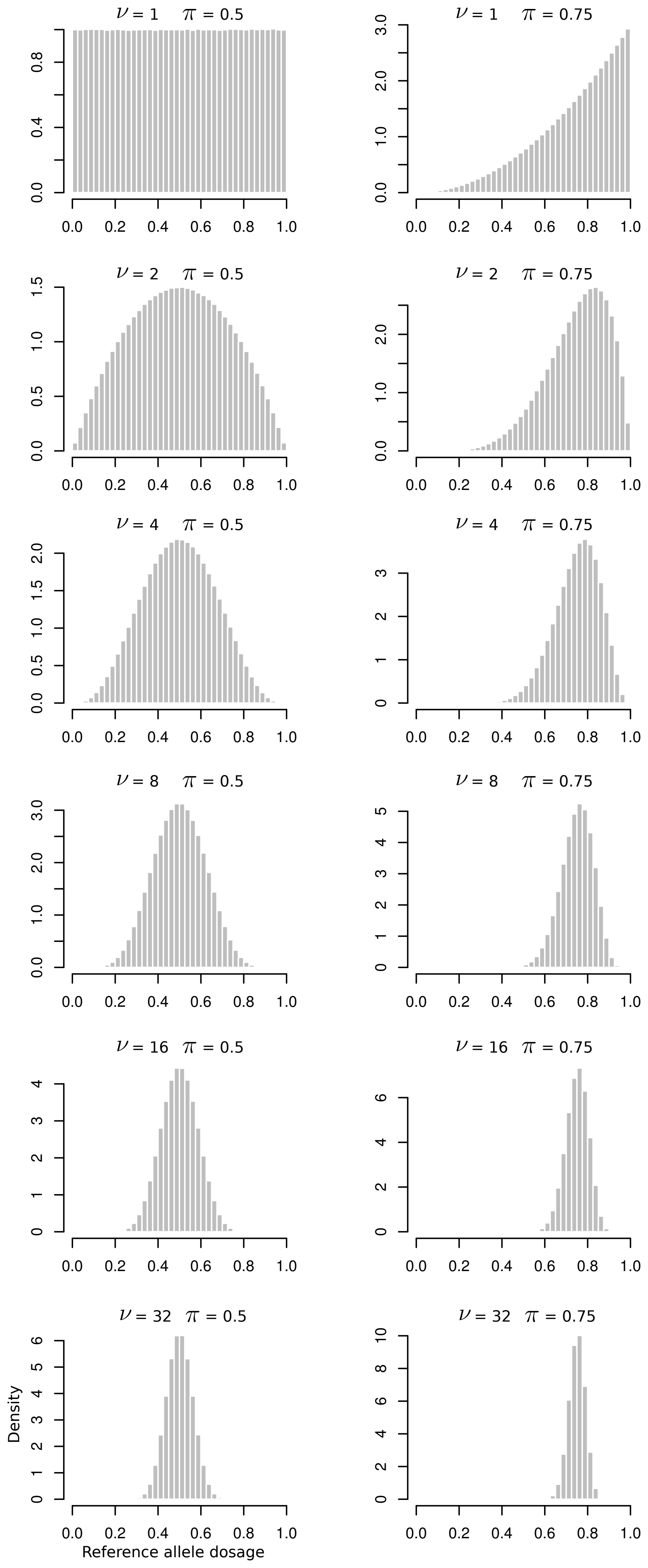

$\nu = 1$ $\pi = 0.5$
$\nu = 1$ $\pi = 0.75$
$\nu = 2$ $\pi = 0.5$
$\nu = 2$ $\pi = 0.75$
$\nu = 4$ $\pi = 0.5$
$\nu = 4$ $\pi = 0.75$
$\nu = 8$ $\pi = 0.5$
$\nu = 8$ $\pi = 0.75$
$\nu = 16$ $\pi = 0.5$
$\nu = 16$ $\pi = 0.75$
$\nu = 32$ $\pi = 0.5$
$\nu = 32$ $\pi = 0.75$
Density
Reference allele dosage
