# Supplementary figures and images for: Parent-progeny imputation from pooled samples for cost-efficient genotyping in plant breeding
Source: PLoS One. 2017 Dec 22;12(12):e0190271. doi: 10.1371/journal.pone.0190271 (PMC5741258; doi:10.1371/journal.pone.0190271)

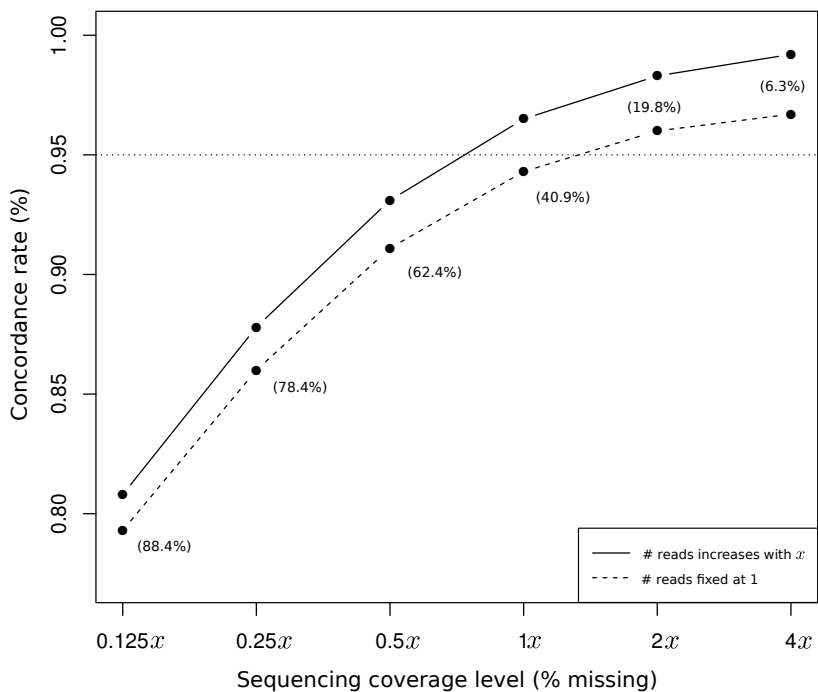

Supplement: S2 Fig — The percent of missing markers (in parentheses) correspond to the expectations at the indicated sequencing coverage levels. The full line shows results from the standard GBS scenario where the read number and % missing loci varies as a function of the sequencing coverage x. Those results are replicated here for comparison purposes. (PDF) [file pone.0190271.s005.pdf]

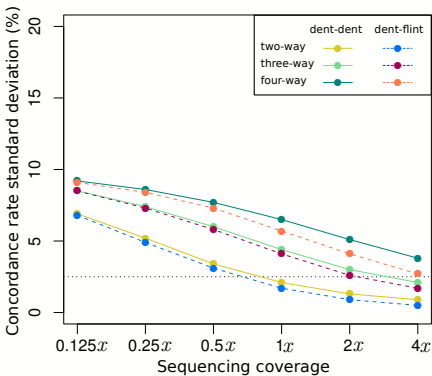

Supplement: S3 Fig — (PDF) [file pone.0190271.s006.pdf]

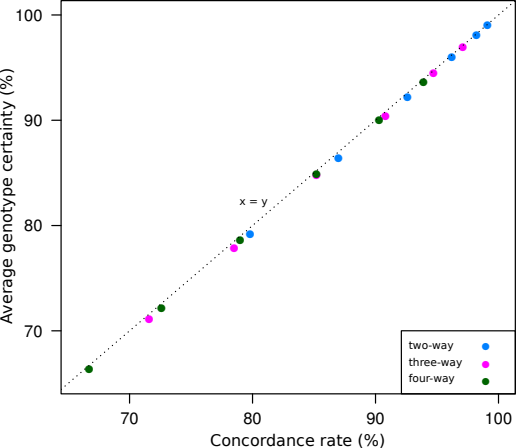

Supplement: S4 Fig — (PDF) [file pone.0190271.s007.pdf]
